# Understanding the Dynamic Loss Modulus of NR/SBR Blends in the Glassy–Rubbery Transition Zone

**DOI:** 10.3390/polym17101312

**Published:** 2025-05-11

**Authors:** Angel J. Marzocca, Marcela A. Mansilla, María Pía Beccar Varela, María Cristina Mariani

**Affiliations:** 1 Laboratorio de Polímeros y Materiales Compuestos, Departamento de Física, Universidad de Buenos Aires, Ciudad Universitaria, Buenos Aires C1428EHA, Argentina; 2 Dirección Técnica de Materiales Avanzados, INTI, CONICET, Av. General Paz 5445, San Martín B1650WAB, Argentina; mmansilla@inti.gob.ar; 3 Department of Mathematical Sciences, University of Texas at El Paso, El Paso, TX 79968, USA; mpvarela@utep.edu (M.P.B.V.); mcmariani@utep.edu (M.C.M.)

**Keywords:** natural rubber, styrene-butadiene rubber, blends, vulcanization, dynamic mechanical properties, glass transition region

## Abstract

The motivation of this research was to analyze the dynamic properties, mainly the loss modulus, of vulcanized immiscible blends of natural rubber (NR) and styrene-butadiene rubber (SBR) in the glass transition zone, where the SBR phase is in a glassy state and the NR phase is in a rubbery state. The blends were cured at 433 and 443 K and studied around the glass transition using a dynamic mechanical analyzer. The dependence of the loss modulus on temperature was described by considering the phase separation, and the frequency dependence was also included to provide a deeper insight into the dynamic properties. This was achieved by integrating the mechanical model proposed by Zener, which considers a single relaxation time related to temperature using both the Arrhenius and Vogel–Fulcher–Tammann (VFT) relations. The best correlation with the data was obtained using the Arrhenius relationship. The activation energy of the NR phase increases with the NR content in the blend, while in the SBR phase, it varies slightly. The trends obtained are related to curative migration from the SBR to the NR phase, increasing the crosslink density at NR domain boundaries. These insights are valuable for optimizing the performance of these elastomeric blends in practical applications.

## 1. Introduction

Natural rubber (NR) and styrene-butadiene rubber (SBR) blends (NR/SBR) are employed in applications that require high technical performance, including tires and conveyor belts. NR exhibits low hysteresis, high elasticity, and a self-reinforcing characteristic resulting from strain-induced crystallization. On the other hand, SBR offers excellent abrasion resistance and reasonably good thermal properties.

NR is not miscible with synthetic rubbers such as SBR. Therefore, the components of the blend are arranged in different domain morphologies depending on many factors, such as the mixing ratio and variation in polymer type and microstructure, as well as polarity, viscosity, and mixing procedure [1,2,3,4,5,6,7,8,9,10,11,12,13,14,15,16,17,18,19,20]. Using atomic force microscopy, Klat et al. [11] observed that domain sizes increased from uncured to fully cured samples at the optimum cure time in a blend of 70 phr NR and 30 phr low-vinyl SBR. Their studies examined blends cured at 413 K and 433 K and found that phase separation was more pronounced at the lower cure temperature.

Having a good model to explain the dynamic mechanical properties of elastomers and elastomeric blends is very important for addressing technological applications. The studies of Klüppel et al. [14], Schuster et al. [15], Wunde and Klüppel [16], and Muller et al. [6] focused on the influence of the phase morphology on energy storage and dissipation during dynamic excitation in unfilled and filled NR/SBR and EPDM/BR blends. The observed, strongly nonlinear, dependence of the local loss modulus maxima on the blend ratio of unfilled blends was explained based on a percolation model that represents a useful framework for modeling the phase network.

Voges et al. [19] investigated NR/SBR blends considering heterogeneous morphologies that consist of regions with nearly pure phases and distinct interphases.

The dynamic mechanical behavior of rubber-like materials is both temperature- and time–frequency-dependent. Information on the changes in dynamic mechanical properties with time or frequency is required in products for engineering applications. Numerous viscoelastic models, namely Cole–Cole, Kohlrausch–Williams–Watts (KWW), Havriliak–Negami (HN), etc., have occasionally been used to describe dynamic mechanical properties. The HN model has a distinct advantage over the other viscoelastic models for its simplicity and ability to accurately predict results [21,22].

For several years, our research group has studied the NR/SBR system using different experimental attacks that include rheometric characterization, swelling, differential scanning calorimetry (DSC), microscopy, dynamic mechanical analysis (DMA), and positron annihilation lifetime spectroscopy (PALS), among others [7,8,9,13,23,24].

In a recent paper, we analyzed the local strains developed in vulcanized NR/SBR blends cured at 433 K and 443 K using sulfur and TBBS (n-t-butyl-2-benzothiazole sulfenamide) as a cure system [13]. The samples were characterized by dynamic mechanical properties between 193 K and 293 K, with interest in the glass transition region of the vulcanized immiscible blends, where the NR and SBR phases are rubbery and glassy, respectively. By studying the loss modulus, this research shows how the local strain in the NR phase varies depending on the amount of SBR in the blend.

This paper presents a new approach to analyzing the loss modulus (*E*) behavior with temperature, within the glass transition region, for cured NR/SBR blends. For a given temperature, the *E* of each elastomer is expressed by a law resulting from the contribution of its amorphous and rubbery structure according to its volume fraction of the glassy phase. Then, the influence of frequency is considered by applying Zener’s mechanical model [25], which assumes a single relaxation time. Finally, the *E* of the blend is presented taking into account the mixing law of the pure elastomers. It is assumed that the morphology and microstructure of each phase depend on the mix composition and curing conditions.

## 2. Materials and Methods

### 2.1. Materials

The compounds studied in this work are composed of NR (SMR-20 (Malaysia)) and SBR-1502 (Arpol (E-SBR) provided by Petrobras (Pto.Gral. San Martin, Argentina)). They were prepared at room temperature by solution blending with the formulation given in Table 1. Details of the sample preparation are given in ref [9]. In the formulation, sulfur (Sigma Aldrich, St. Louis, MO, USA) and TBBS (n-t-butyl-2-benzothiazole sulfenamide) (Vulkacit, NZ/EG-C, Lansexx, Germany) were used as the cure system. The accelerator/sulfur ratio, *Λ*, is 1; therefore, this cure system is semi-EV [26]. Stearic acid (Sigma Aldrich, St. Louis, MO, USA) and zinc oxide (Sigma Aldrich, St. Louis, MO, USA) are activators of the curing reaction. From the rheometer curves at 433 K and 443 K, the optimum cure time *t*_100_ (time to reach the maximum degree of cure) was obtained for each sample. The values are summarized in Table 2 for each compound and cure temperature. The onset of cure, defined as the time of 5 percent conversion (*t*_5_), for each compound is also shown in Table 2.

All samples were cured at 433 K and 443 K at their respective *t*_100_ times, using a hydraulic press set at 5 MPa. The compounds were molded into sheets with dimensions of 50 × 40 × 2 mm^3^. After the curing process, the samples were immediately cooled in an ice–water mixture.

### 2.2. Dynamic Mechanical Tests

Dynamic mechanical analysis (DMA) measurements were performed using a dynamic mechanical analyzer (Gabo Qualimeter (Hannover, Germany), model Eplexor 500N). Details of the measurements performed can be found in ref [13].

### 2.3. Methodology

In pure elastomeric compounds, for example, NR or SBR, there is a temperature range (the glass transition region) where glassy and rubbery phases coexist. They have separate contributions to the loss modulus, and the upper bound, known as the Reuss limit, is reached in the limiting case of a homogenous distribution of the strain; this can be proposed as [27]

(1) $$E^{\prime \prime }=\upsilon_{g}{E^{\prime \prime }}_{g}+\left(1-\upsilon_{g}\right){E^{\prime \prime }}_{a}$$

where ${E^{\prime \prime }}_{a}$ and ${E^{\prime \prime }}_{g}$ are the loss moduli of the rubbery and glassy phases, respectively, and $\upsilon_{g}$ is the volume fraction of the glassy phase.

A simple methodology is proposed in this paper to estimate υ_g_ from the loss modulus plot when it changes with temperature T in an isochronous state (at a fixed frequency).

**Figure 1:**
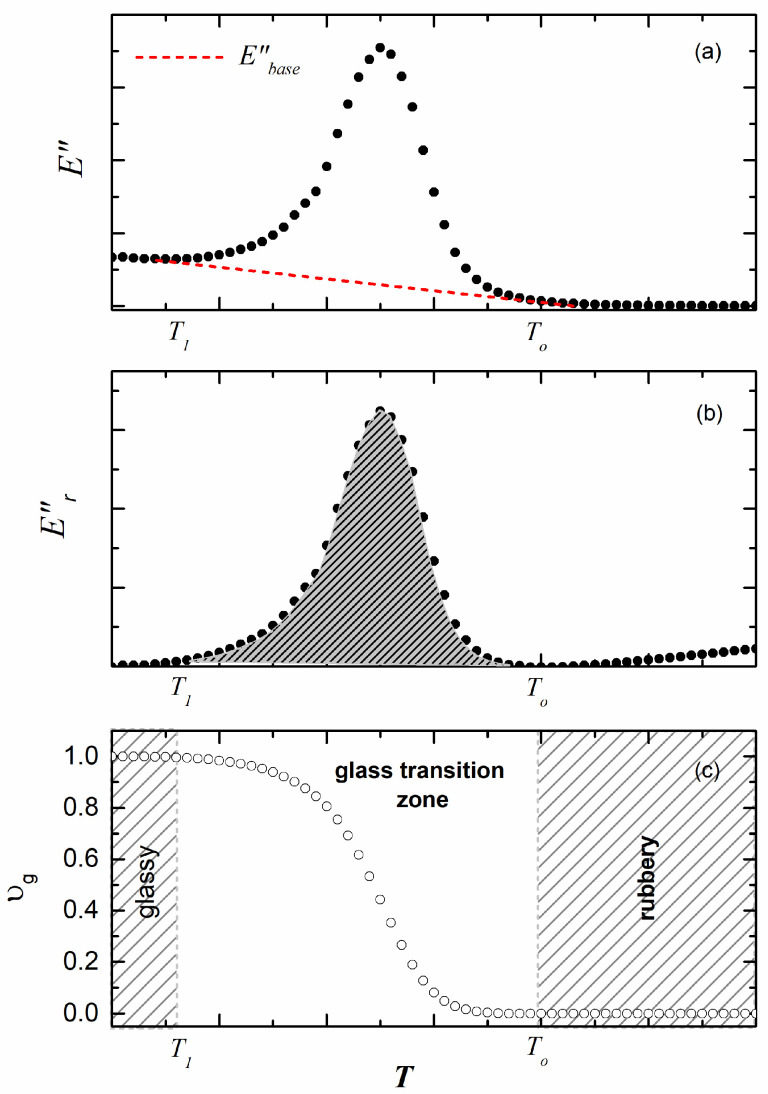
(**a**) Loss modulus *E* as a function of temperature *T*. (**b**) $E_{r\;}^{\prime \prime }$ as a function of temperature *T*. (**c**) $\upsilon_{g}$ as a function of temperature *T* obtained by Equation (3).

Figure 1a shows a typical loss modulus of an elastomer as it changes from a rubbery to a glassy state with decreasing temperature. In this type of plot, a baseline $E_{base}^{\prime \prime }(T)$ is defined between the temperatures *T*_0_ and *T*_1_ (shown in Figure 1a). The resultant loss modulus can be introduced as

(2) $$E_{r}^{\prime \prime }(T)=E^{\prime \prime }(T)-E_{base}^{\prime \prime }(T)$$

which is shown in Figure 1b. The dashed region in Figure 1b is the integral between *T*_0_ and *T*_1_.

In the next step, $\upsilon_{g}$, the variation in the normalized integral of $E_{r\;}^{\prime \prime }\left(T\right)$ as a function of the temperature is calculated as

(3) $$\upsilon_{g}(T)=\frac{\int_{T_{o}}^{T}E_{r\;}^{\prime \prime }\left(T\right)dT}{\int_{T_{0}}^{T_{1}}E_{r\;}^{\prime \prime }\left(T\right)dT}$$

This correlation is depicted in Figure 1c as a function of temperature. In this analysis,

$${\;\;\upsilon }_{g}=0\;\;\;\;\;\;\;\;\;\;\;\;T>T_{o}\;\;\;\;\mathrm{rubbery}\mathrm{zone}$$

$$\upsilon_{g}\;\;\;\;\;\;\;\;\;\;\;\;\;\;T_{1}<T<T_{o}\;\;\;\;\mathrm{glass}\mathrm{transition}\mathrm{zone}$$

$$\upsilon_{g}=1\;\;\;\;\;\;\;\;\;\;\;T<T_{1}\;\;\;\mathrm{glass}\mathrm{zone}$$

By normalizing Equation (3), we can remove any scaling effects and focus purely on the temperature-dependent behavior. The temperature dependence of $\upsilon_{g}$ is a key factor in the behavior of elastomers, especially when it comes to how the proportions of the glass phase within the material change, affecting its overall mechanical behavior.

The Boltzmann equation, often represented by a sigmoid curve, is commonly used to describe the transition of a dependent variable from one state to another, typically in relation to an independent variable. In this context, the Boltzmann sigmoidal equation can be used to model the transition of a property, such as the glassy volume fraction, $\upsilon_{g}$, as a function of temperature. By fitting experimental data to this equation, we can gain insights into the underlying physics driving the transition phenomena in the elastomers.

Due to the structural and morphological heterogeneity of semi-crystalline polymers and their blends, simultaneous double crystallization processes are common [28].

In the case of an isochronous process in a DMA, performing tests at a constant frequency while varying the temperature, an empirical double Boltzmann function can be introduced as

(4) υg=∑i=12fi1−expT−Ti/ki∑i=12fi=1

If *f = f*_1_, then *f*_2_ = (1 − *f*_1_), and by replacing it in Equation (4), the following relationship is obtained:

(5) $$\upsilon_{g}=\left(\frac{f}{1-exp\left(\left(T-T_{1}\right)/k_{1}\right)}+\frac{\left(1-f\right)}{1-exp\left(\left(T-T_{2}\right)/k_{2}\right)}\right)$$

where *k*_1_ and *k*_2_ are the constant intervals that control the rise in phase 1 and phase 2 (also called slope factors).

It is known that temperature-induced crystallization (TIC) is a process that occurs in NR [29]. The rate of crystallization depends on the temperature and duration of crystallization. This factor can influence the size and number of crystallites with a random orientation. For TIC samples, both amorphous chains and crystallites are present. The process creates a wide distribution of crystallite sizes because the crystallization process occurs under static conditions where random regions are crystallized [29]. Equation (5) proposes that, in principle, two processes govern crystallization. This is a simplified way of analyzing the problem, and the relationship is established empirically.

The dependence of the loss modulus *E* on the frequency, based on the mechanical model proposed by Zener for a single relaxation time, has the relationship [25]

(6) $$E^{\prime \prime }=\frac{∆E\;\omega \tau }{1+\omega^{2}\tau^{2}}$$

with the relaxation intensity

(7) $$∆E=\left(E_{u}-E_{r}\right)$$

where *E_u_* is the unrelaxed modulus and *E_r_* is the relaxed modulus; *ω* is the angular frequency; and τ is the relaxation time of the process.

The α-relaxation in polymers associated with the glass transition has been analyzed using various models in the literature. Among these are the free volume theory [30], the Adams–Gibbs theory [31,32], the coupling mode theory [33], the coupling model [34,35], and atomistic simulations [36], among others. The Adam–Gibbs theory provides the theoretical foundation for the Vogel–Fulcher–Tammann (VFT) equation [37,38,39], which is widely regarded as an accurate representation of the temperature dependence of the relaxation time τ. It is expressed as

(8) $$\tau =Aexp\left[\frac{B}{T-T_{v}}\right]$$

where *A* is a hypothetical relaxation time at infinite temperature, *B* is a fitted parameter that is sometimes related to fragility, *T* is the absolute temperature, and *T_V_* is the Vogel temperature that is often considered the temperature that is reached upon quasi-static cooling, at which chain segments become immobile. *T_V_* is occasionally associated with an “ideal” glass transition, typically occurring 30–70 K below *T_g_* [37,38,39].

It is also quite common to find a dependence between *τ* and temperature that follows an Arrhenius relationship of the form [18,24,40,41,42,43]

(9) $$\tau =\tau_{o}exp\left[\frac{H_{ac}}{RT}\right]$$

where *H_a_*_c_ is the activation energy of the single process, *τ_o_* is a constant, and R is the gas constant (8.314 J/mol K).

Considering the contributions of Equations (1) and (6), the following relationship can be proposed for the loss modulus:

(10) $$E^{\prime \prime }={E^{\prime \prime }}_{g}\upsilon_{g}+{E^{\prime \prime }}_{a}\left(1-\upsilon_{g}\right)+\frac{\Delta E\omega \tau }{1+\omega^{2}\tau^{2}}$$

This equation assumes that the loss modulus behavior with frequency and temperature follows the Zener model (expressed by Equation (6)), but it adds a thermal background resulting from the structural change as the compound passes from the rubbery phase to the glassy phase as the temperature decreases (in the glass transition region).

In the context of immiscible blends of two components with loss moduli $E_{I}^{\prime \prime }$ and $E_{II}^{\prime \prime }$, respectively, and volume fractions *ϕ_I_* and *ϕ_II_*, the loss modulus of the blend $E_{blend}^{\prime \prime }$ can be analyzed by introducing a mixture law along with an additional term ${E\prime \prime }_{ex}$ [14]. This term accounts for the presence of an interface characterized by properties that differ from those of the individual components. Then,

(11) $$E_{blend}^{\prime \prime }=\phi_{I}E_{I}^{\prime \prime }+\phi_{II}E_{II}^{\prime \prime }+{E^{\prime \prime }}_{ex}$$

In an immiscible blend where one domain is mainly in the rubbery state and the other one is changing from rubbery to glassy as the temperature decreases (this happens in NR/SBR blends in the glass transition region), we can analyze what happens when the interface term is small compared to the mixing law.

The interaction expressed by ${E^{\prime \prime }}_{ex}$ can be disregarded, and an attempt can be made to fit the experimental data using only the mixing law. It must be stressed that this solution is only an estimate and deviations may require the addition of this term.

Therefore, first-order analysis is conducted considering the following relationship:

(12) $$E_{blend}^{\prime \prime }\;\approx \;\phi_{I}E_{I}^{\prime \prime }+\phi_{II}E_{II}^{\prime \prime }$$

Then, considering that Equation (10) represents the loss modulus of each component, a relationship to describe the case of blends is proposed as a mixture law:

(13) $$E_{blend}^{\prime \prime }=\phi_{I}\left[{E^{\prime \prime }}_{g,I}\upsilon_{g,I}+{E^{\prime \prime }}_{a,I}\left(1-\upsilon_{g,I}\right)+\frac{{\Delta E}_{I}\omega \tau_{I}}{1+\omega^{2}\tau_{I}^{2}}\right]+\phi_{II}\left[{E^{\prime \prime }}_{g,II}\upsilon_{g,}+{E^{\prime \prime }}_{a,II}\left(1-\upsilon_{g,II}\right)+\frac{{\Delta E}_{II}\omega \tau_{II}}{1+\omega^{2}\tau_{II}^{2}}\right]$$

The relaxation times $\tau_{I}$ and $\tau_{II}$ depend on whether the model used is VFT (Equation (8)) or Arrhenius (Equation (9)). In the first case, the parameters involved are *A_I_*, *A_II_*, *B_I_*, *B_II_*, *T*_*V*,*I*_, and *T*_*V*,*II*_, and in the second case, $\tau_{o,I}$, $\tau_{o,II}$, *H*_*ac*,*I*_, and *H*_*ac*,*II*_.

## 3. Results and Discussion

**Figure 2:**
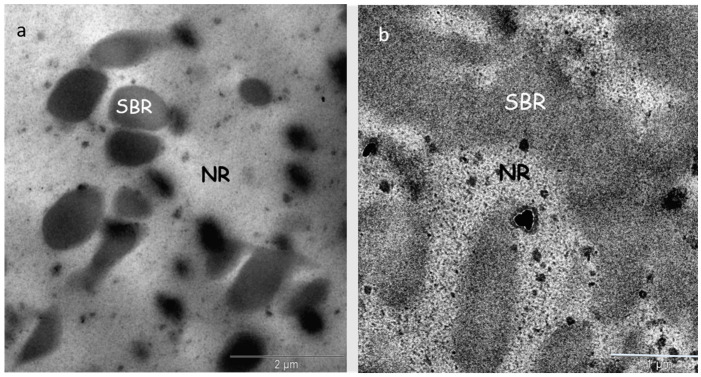
TEM micrograph of NR70/SBR30 (**a**) and NR50/SBR50 (**b**) blends. SBR is the dark phase and NR is the clear phase.

NR does not mix homogeneously with synthetic rubbers such as SBR, resulting in the formation of distinct domain morphologies within the blend. As an example, Figure 2 presents the microstructure of the 70NR/30SBR and NR50/SBR50 blends cured at 433 K used in this study, as observed through TEM (Philips CM200 (200 kV)). Heterogeneity is evident in the sample, with the NR and SBR phases distinctly visible.

The NR and SBR areas were calculated in both images using ImageJ software 1.53t. For the NR75/SBR25 blend (Figure 2a), the percentages of NR and SBR were 75.1% and 24.9%, respectively. The blend shows a sea–island structure with small SBR droplets in the NR matrix. The SBR domains are nearly spherical, with a most probable diameter of around 0.46 um.

Regarding the NR50/SBR50 blend (Figure 2b), which displays a nearly co-continuous shape, the percentage of NR was determined to be 48.1%, while the percentage of SBR was found to be 51.9%.

In previous studies of the NR/SBR blend used in the present research, the NR and SBR phases were observed by optical microscopy and TEM [8,22]. These findings align with other results reported in the literature [3,10,11,12].

**Figure 3:**
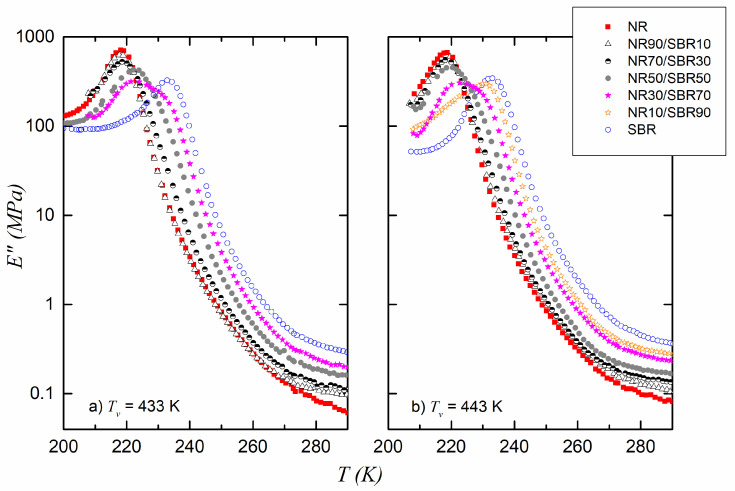
Loss modulus E as a function of the test temperature for pure compounds and blends cured at (**a**) T_v_ = 433 K and (**b**) T_v_ = 443 K. Data from ref [13].

Figure 3a,b show the loss modulus of the compounds cured at 433 K and 443 K. The variation in E as a function of temperature can be used to make a first estimate of the glassy volume fraction change of the pure elastomer compounds (NR and SBR) as they pass through the glass transition region. Although some of these measurements were replicated, no significant differences were found between them that would warrant placing error bars in the figures (the instrumental error was also very small).

**Figure 4:**
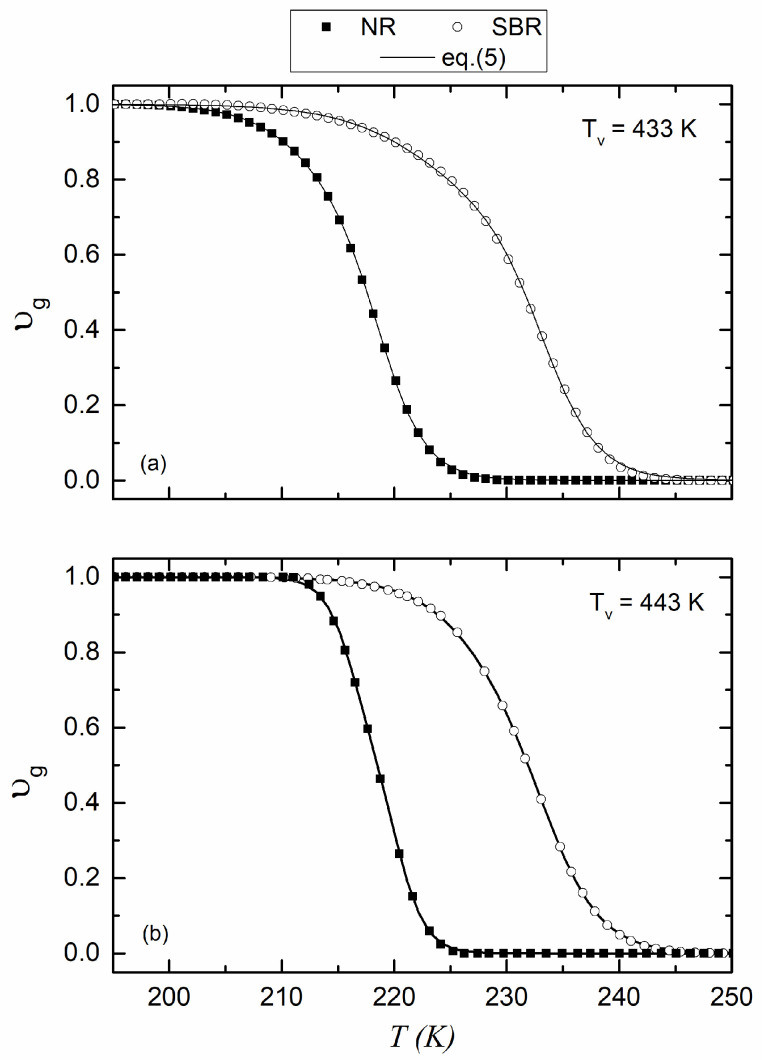
Volume fraction of the glassy phase as a function of the temperature of NR and SBR vulcanized at 433 K (**a**) and 443 K (**b**). Solid lines are the fitting to Equation (5).

Based on Figure 3a,b and using Equation (3), $\upsilon_{g}$ is calculated, and its temperature variation is presented in Figure 4a,b for NR and SBR vulcanized at 433 K and 443 K, respectively.

The data from Figure 4a,b were then fitted using the double Boltzmann function described in Equation (5), resulting in an excellent fit, as evidenced by the continuous line shown in both figures. The optimal parameters obtained from the fitting are provided in Table 3, along with the R*^2^* coefficient.

**Figure 5:**
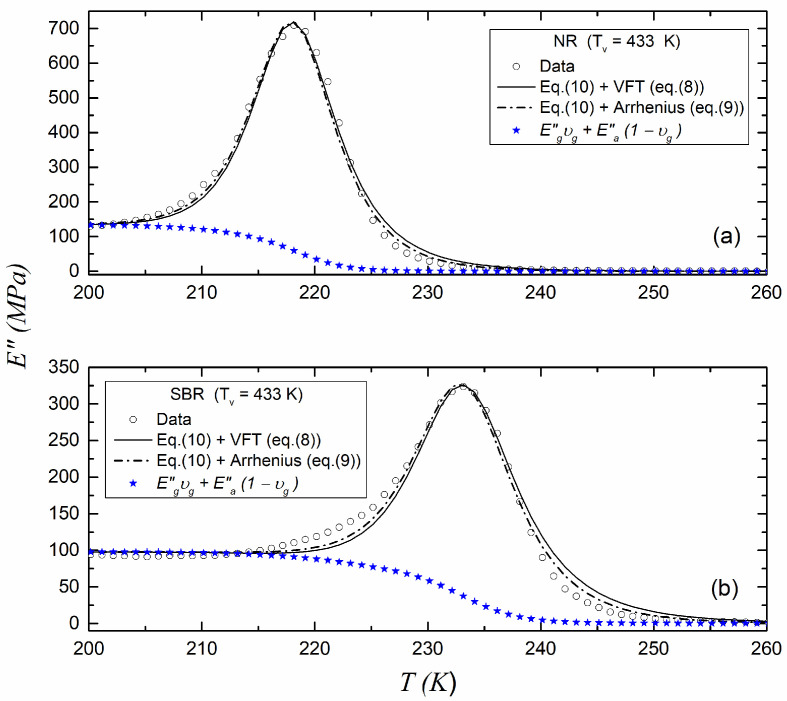
Loss modulus E for (**a**) NR and (**b**) SBR vulcanized at 433 K with fitting to Equation (10).

**Figure 6:**
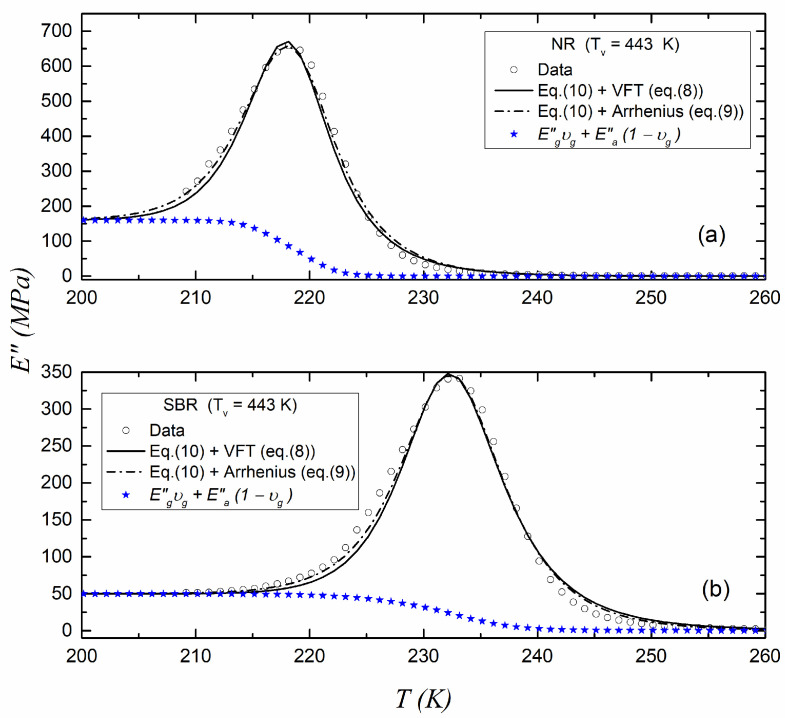
Loss modulus E for (**a**) NR and (**b**) SBR vulcanized at 443 K with fitting to Equation (10).

Figure 5 and Figure 6 show the fitting of the experimental data of E as a function of the temperature using Equation (10) in the glass transition region for the NR and SBR samples cured at 433 K and 443 K, respectively. In the figures, both the VFT (Equation (8)) and Arrhenius (Equation (9)) expressions have been used for the relaxation time in Equation (10). The parameters used for fitting the data are given in Table 4. The contribution of Equation (1) is also shown in the figures.

Table 4 shows the parameter R^2^ obtained by fitting the experimental data to Equation (10). It can be observed that when considering the Arrhenius relation for the relaxation time included in Equation (10), the best R^2^ was always obtained, regardless of the vulcanization temperature of the samples. Therefore, we decided to use the Arrhenius relationship instead of the VFT relationship in Equation (10) for the remaining fits of the experimental data of E of the vulcanized blends.

**Figure 7:**
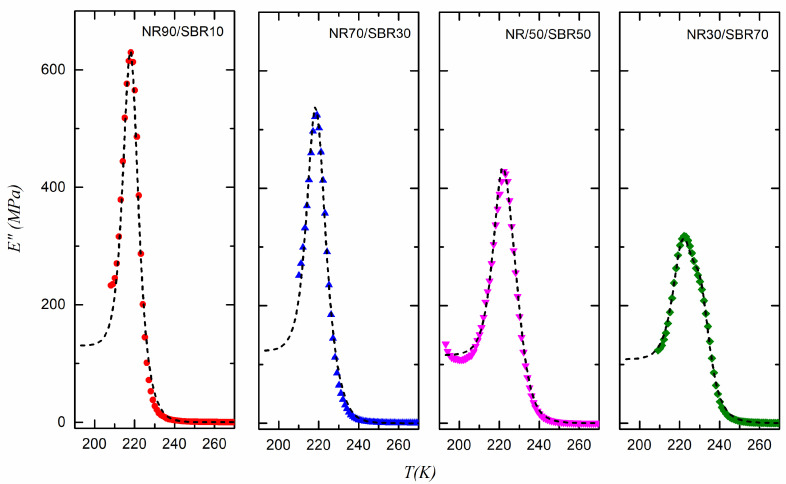
E for NR/SBR blends vulcanized at 433 K. Dashed line corresponds to the fitting to Equation (13).

**Figure 8:**
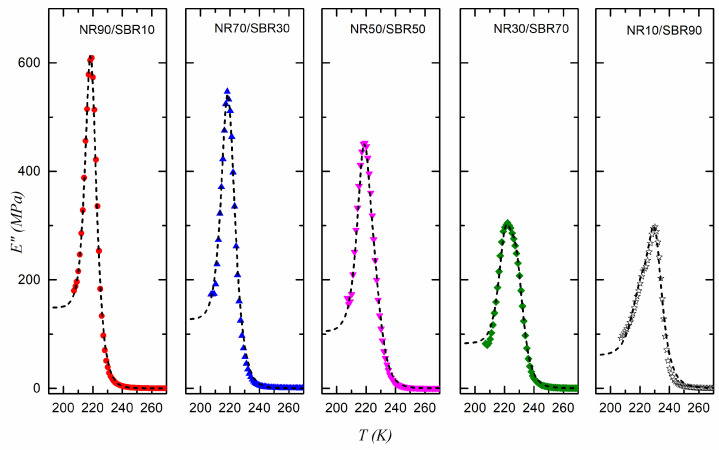
E for NR/SBR blends vulcanized at 443 K. Dashed line corresponds to the fitting to Equation (13).

**Figure 9:**
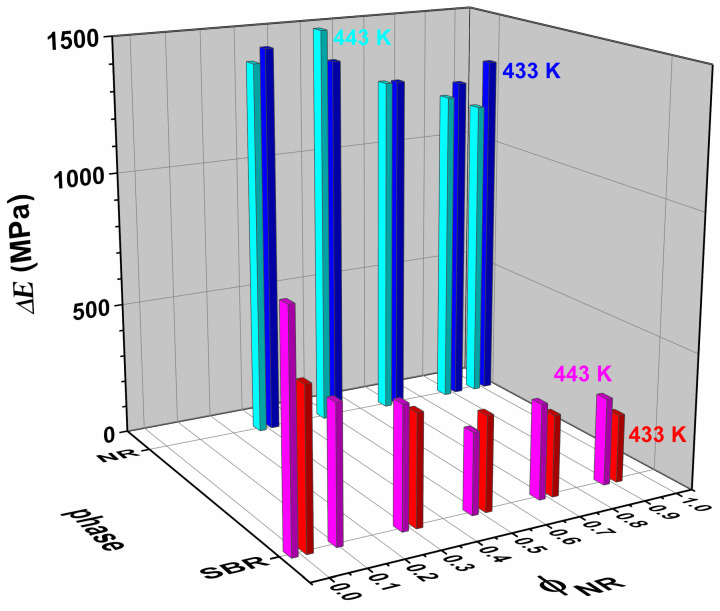
ΔE for NR and SBR phases as a function of the NR content, $\phi_{NR}$ in the blends vulcanized at 433 K and 443 K.

**Figure 10:**
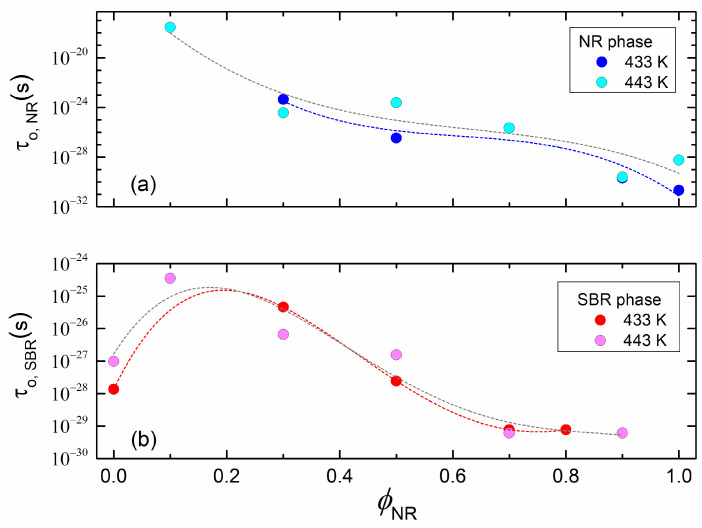
*τ*_o,NR_ (**a**) and *τ*_o,SBR_ (**b**) for the pure compounds and each phase of the blends vulcanized at 433 K and 443 K. Dashed lines are included to show data tendency.

**Figure 11:**
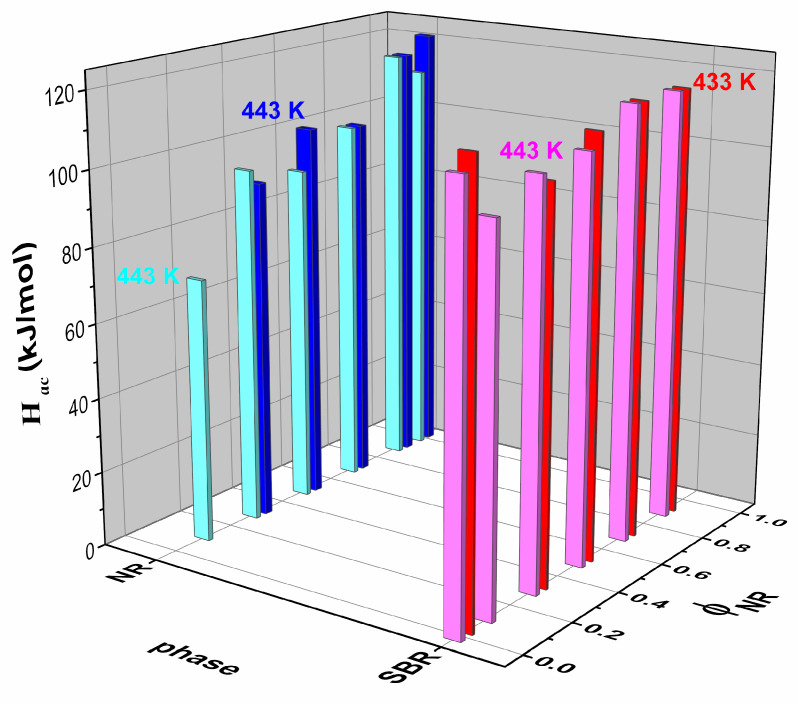
$H_{ac}$ for NR and SBR phases as a function of the NR content, $\phi_{NR}$, in the blends vulcanized at 433 K and 443 K.

The mixing law proposed in Equation (13) can be used to represent the experimental loss modulus data obtained for the different NR/SBR blends prepared, where phase I = NR and phase II = SBR. Figure 7 and Figure 8 show the plots of the data for the blends vulcanized at 433 K and 443 K, together with the fitted curves. The parameters of Equation (13) (considering Equation (9) as the relaxation time) that best fit the experimental data are shown in Figure 9, Figure 10 and Figure 11 for the samples vulcanized at 433 K and 443 K. These figures show how these parameters change with the volume fraction, *ϕ*(NR), of NR in the vulcanized blends.

Figure 9 shows the relaxation intensity, ∆E, for the NR and SBR phases, as a function of the NR content in the blend $\phi_{NR}$ for the samples cured at 433 K and 443 K. In the case of the NR phase, ${∆E}_{NR}$ tends to decrease as the blend is richer in NR. This behavior is observed for both curing temperatures, and it can be associated with the curative migration among phases.

In the present study, mapping of the distribution of curatives into the phases of the blends, as presented in the work of Cosa Fernandez et al. in NR/SBR mixtures [44], has not been carried out. The phenomenon of the migration of curatives (mainly sulfur and accelerators) has also been verified indirectly in NR/SBR blends [7,23,24,45,46]. Migration occurs from the BR or SBR phase toward the NR phase and results in a higher concentration of curatives in the NR phase, which leads to a change in the crosslinking density. As a result of this effect, there is a temperature shift in the glass transition temperature of each phase of the blend [46].

Figure 9 also shows the relaxation intensity for the SBR phase, ${∆E}_{SBR}$, as a function of $\phi_{NR}$ in the blend. As the $\phi_{NR}$ increases, the reduction in the relaxation intensity of the SBR phase is more significant. From the observation of t_100_ in each compound (Table 1), it can be concluded that in the most NR-rich blends, these times would be insufficient to achieve the development of a complete crosslinked network in the SBR phase, and therefore this phase is undervulcanized.

The trend with $\phi_{NR}$ is similar at both vulcanization temperatures used in this research for ${∆E}_{NR}$ and ${∆E}_{SBR}$. In the case of the pure NR compound ($\phi_{NR}=1)$, ${∆E}_{NR}$ is lower when the sample is cured at 443 K compared to 433 K. For the pure SBR compound ($\phi_{NR}=0)$, this situation is reversed.

When the blends are analyzed, it is observed that the relaxation intensity of each phase of the sample NR50/SBR50 presents a different tendency with the cure temperature compared to the other ones. For the other blends, ${∆E}_{NR}$ is higher or equal for the samples cured at 433 K compared to those cured at 443 K, but the opposite situation is observed for ${∆E}_{SBR}$.

Using the definition of the relaxation intensity in Equation (7), it is the difference between the unrelaxed and relaxed moduli. The unrelaxed modulus, $E_{u}$ (associated with the glassy zone), does not change too much with the network formed during the crosslinking process. The observed stability can be attributed to the fact that, within the glassy zone, the material’s stiffness is predominantly determined by the intrinsic properties of the polymer chains themselves, rather than by the crosslink density. However, the relaxed modulus, $E_{r}$ (associated with the rubbery zone), is more sensitive to the type of network structure formed in both phases and it depends on the curing temperature.

The parameters τ_o,NR_ and τ_o,SBR_ were also estimated by fitting the data from Figure 7 and Figure 8 to the proposed model described by Equation (13). Figure 10a,b show the dependence of these parameters on $\phi_{NR}$ and the cure temperature in the studied compounds for the NR (a) and SBR (b) phases. It is observed that both parameters decrease when the blend becomes richer in NR. However, it can also be observed that when a small amount of NR is added to the pure SBR compound, τ_o,SBR_ increases initially but starts to decrease as the NR content $\phi_{NR}$ continues to rise.

Figure 11 shows the activation energy for the NR phase ($H_{ac,NR}$) as a function of the NR content in the blend. The trend shows that $H_{ac,NR}$ increases as the NR content increases, regardless of the sample cure temperature. The monotonic increase in activation energy with higher $\phi_{NR}$ for both cure temperatures indicates that the NR phase becomes more thermally stable or requires more energy to undergo molecular motion, implying that the network structure in the NR phase becomes more constrained. The crosslink density in the NR phase may increase with higher NR content, contributing to this effect. The migration of curatives from the SBR to the NR phase during the vulcanization process can indeed explain the observed trends in the activation energy for both phases due to the fact that this migration increases the crosslink density in the NR phase [45,46].

As mentioned previously, the gradual increase in the NR phase in the SBR matrix alters the values of t_100_ obtained in the rheometer test (Table 1). From these values, it is evident that, at both curing temperatures of 433 K and 443 K, the addition of just 10 phr of NR significantly reduces t_100_. In this case, the SBR phase is likely not fully cured, while the NR phase is overcured, as indicated by the lower t_100_ values of the pure NR compound. The presence of more interfaces in SBR as $\phi_{NR}$ increases, combined with the migration of curatives into the NR phase, likely contributes to the rise in activation energy. Further investigation must be carried out to elucidate this point.

On the other hand, Figure 11 also presents the activation energy for the SBR phase ($H_{ac,SBR}$) as a function of $\phi_{NR}$ in the blend. Initially, $H_{ac,SBR}$ is approximately 115 kJ/mol for the pure SBR compound ($\phi_{NR}=0)$, and decreases slightly with the addition of NR to the blend. However, for $\phi_{NR}$ > 0.3, this trend reverses and $H_{ac,SBR}$ begins to increase.

In previous research, our research group employed a sub-resonant forced pendulum to measure the loss tangent in the glass transition region, determining the activation energy for NR/SBR blends cured at 433 K [24]. The values were similar to those in the present study. Although these compounds were also prepared via solution casting, the curing system employed was the CV type with a Λ value of 0.31. In that study, $H_{ac,NR}$ and $H_{ac,SBR}$ exhibited the same trend, showing slightly higher values as $\phi_{NR}$ increased in the blend. This observation suggests that the curing system influences the activation energy, which is reasonable, as it likely results in a different type and distribution of crosslinks. Further investigation must be carried out to elucidate this point.

## 4. Conclusions

In this research, we have analyzed the variation in the loss modulus with temperature in unfilled NR/SBR composites cured at 433 K and 443 K. The studies focused on the glass transition region. The samples were prepared at their optimal curing conditions by vulcanizing them at time t_100_ obtained by means of rheometry.

As extensively reported in the literature, these types of blends are immiscible, and we have confirmed this through our TEM observations.

A new approach to fitting loss modulus data as a function of the temperature in the glass transition region, obtained by DMA, is introduced and validated. This methodology takes account of the coexistence of the rubbery and glassy phases of the pure elastomer as the temperature transitions between the rubbery and glassy states (and vice versa). In the analysis, the temperature and frequency dependence of the loss modulus is considered, based on Zener’s mechanical model for a single relaxation time.

This methodology was successfully applied to the case of an immiscible blend, namely cured unfilled NR/SBR, where a mixture law for both elastomers was considered.

This analysis yielded key model parameters—activation energy, intensity, and relaxation time—for each phase within the blends, highlighting how these parameters shift as the NR content increases in the blend. This reveals how the properties of each phase in the blend vary according to the blend composition.

## Figures and Tables

**Table 1:** Blend formulations.

	SBR	NR10/SBR90	NR30/SBR70	NR50/SBR50	NR70/SBR30	NR90/SBR10	NR
NR (SMR20)	0	10	30	50	70	90	100
SBR-1502	100	90	70	50	30	10	0
Stearic Acid	2						
Zinc Oxide	5						
Sulfur	1.5						
TBBS	1.5						

**Table 2:** Cure times (*t*_5_ and *t*_100_) at 433 K and 443 K obtained from rheometer tests [13].

*T*_*v*_ (K)	*t* (min)	SBR	NR10/SBR90	NR30/SBR70	NR50/SBR50	NR70/SBR30	NR90/SBR10	NR
433	*t*_5_	13.09	7.91	8.61	5.56	4.14	0.74	0.57
								0.5
								0.57
	*t*_100_	88.30	48.60	46.7	33.50	21.60	14.70	13.70
443	*t*_5_	5.35	3.45	3.26	2.80	0.65	0.55	0.40
	*t*_100_	34.40	25.80	22.10	17.40	11.40	7.00	7.70

**Table 3:** Fitting parameters of Equation (5) and R^2^ coefficient for data shown in Figure 4a,b, for NR and SBR vulcanized at 433 K and 443 K.

	NR		SBR	
*T_v_* (K)	433	443	433	443
*f*	0.28 ± 0.07	0.46 ± 0.05	0.32 ± 0.04	0.41 ± 0.12
T_1_ (K)	212.62 ± 1.14	216.30 ± 0.20	223.62 ± 0.92	228.28 ± 1.13
T_2_ (K)	218.65 ± 0.14	220.24 ± 0.17	233.27 ± 0.11	233.60 ± 0.28
k_1_ (K)	3.17 ± 0.19	1.32 ± 0.06	4.36 ± 0.22	3.44 ± 0.11
k_2_ (K)	2.06 ± 0.08	1.32 ± 0.05	2.39 ± 0.08	2.42 ± 0.14
R^2^	0.99995	0.99992	0.9999	0.99997

**Table 4:** Fitting parameters of Equation (9) for data shown in Figure 5 and Figure 6 using the VFT and the Arrhenius approaches for the relaxation time in Equation (10).

		NR		SBR	
	*T_v_* [K]	433	443	433	443
	$E_{a}^{\prime \prime }\;$ [MPa]	0.056	0.076	0.281	0.35
	$E_{g}^{\prime \prime }\;$ [MPa]	133.9	160	98	50
VFT Equation (8)	*A* [s]	3.9 × 10^−16^	4.0 × 10^−16^	1.0 × 10^−13^	0.8 × 10^−13^
	B [K]	3260	3250	2630	2640
	Tv [K]	113.9	114.5	131.5	131.0
	*R^2^*	0.9493	0.9153	0.9367	0.9166
Arrhenius Equation (9)	*τ_o_* [s]	2.14 × 10^−31^	5.75 × 10^−29^	1.36 × 10^−28^	9.73 × 10^−28^
	*H_ac_* [kJ/mol]	120.6	110.6	116.4	112.2
	*R^2^*	0.9543	0.9438	0.9568	0.9436

## Data Availability

The original contributions presented in this study are included in the article. Further inquiries can be directed to the corresponding author.
